# Pancreatic Enzyme Replacement Therapy in Patients Undergoing First-Line Gemcitabine Plus *nab*-paclitaxel for Advanced Pancreatic Adenocarcinoma

**DOI:** 10.3389/fonc.2021.688889

**Published:** 2021-09-09

**Authors:** Ilaria Trestini, Luisa Carbognin, Umberto Peretti, Isabella Sperduti, Alberto Caldart, Daniela Tregnago, Alice Avancini, Alessandra Auriemma, Giulia Orsi, Sara Pilotto, Luca Frulloni, Gabriele Capurso, Emilio Bria, Michele Reni, Giampaolo Tortora, Michele Milella

**Affiliations:** ^1^Section of Oncology, Department of Medicine, University of Verona, Azienda Ospedaliera Universitaria Integrata (AOUI) di Verona, Verona, Italy; ^2^Division of Gynecologic Oncology, Department of Woman and Child Health and Public Health, Fondazione Policlinico Universitario Agostino Gemelli IRCCS, Rome, Italy; ^3^Biomedical Sciences, Department of Medicine, University of Verona, Verona, Italy; ^4^Department of Medical Oncology, IRCCS San Raffaele Scientific Institute, Milan, Italy; ^5^Biostatistics Unit, IRCCS Regina Elena National Cancer Institute, Rome, Italy; ^6^Department of Medicine, University of Verona, Verona, Italy; ^7^Pancreato-Biliary Endoscopy and Endosonography Division, IRCCS San Raffaele Scientific Institute, Milan, Italy; ^8^Comprehensive Cancer Center, Unità Operativa Complessa (UOC) Medical Oncology, Fondazione Policlinico Universitario Agostino Gemelli IRCCS, Università Cattolica Del Sacro Cuore, Rome, Italy

**Keywords:** pancreatic enzyme replacement therapy, advanced pancreatic adenocarcinoma, survival, maldigestion, weight gain

## Abstract

**Background:**

The clinical consequences of pancreatic exocrine insufficiency and its treatment in advanced pancreatic ductal adenocarcinoma (PDAC) are poorly investigated. This retrospective study aims at investigating the pancreatic enzyme replacement therapy (PERT) use and its impact on survival and maldigestion-related symptoms in advanced PDAC patients undergoing chemotherapy.

**Methods:**

A retrospective analysis was conducted on advanced PDAC patients, treated with first-line gemcitabine plus *nab*-paclitaxel at two academic institutions (March 2015-October 2018). Data were correlated with overall survival (OS) using Cox regression model. Kaplan-Meier curves were compared using Log-Rank test.

**Results:**

Data from 110 patients were gathered. PERT was administered in 55 patients (50%). No significant differences in baseline characteristics with those who did not receive PERT were found. Median OS for the entire group was 12 months (95% CI 9-15). At multivariate analysis, previous surgical resection of the primary tumor, (HR 2.67, *p=0.11*), weight gain after 3 months (HR 1.68, *p=0.07*) and PERT (HR 2.85, *p ≤ 0.001*) were independent predictors of OS. Patients who received PERT reported an improvement of maldigestion-related symptoms at 3 months more frequently than patients who did not (85.2% vs 14.8%, *p ≤ 0.0001*).

**Conclusion:**

PERT is associated with significantly prolonged survival and maldigestion-related symptoms alleviation in advanced PDAC patients.

## Introduction

Pancreatic ductal adenocarcinoma (PDAC) arguably represents the most lethal solid malignancy; its incidence is increasing worldwide and it is expected to become the second cause of cancer-related death by 2030 (1, 2). Symptoms are usually non-specific, diagnosis is often delayed, and most patients present with locally advanced or metastatic disease at the time of diagnosis, which makes them not candidate for surgery (1). Furthermore, a considerable proportion of patients do not receive any treatment, due to advanced age, comorbidities, and compromised clinical conditions (3). Particularly, unintentional weight loss and nutritional deterioration, common presentation hallmarks among advanced PDAC patients, may contribute to reducing a person’s ability to receive chemotherapy, thereby impairing their survival expectations (4).

The major causes of malnutrition in PDAC patients are cancer-induced metabolic changes, triggered by a complex network of pro-inflammatory catabolic cytokines and another tumor- and host-derived humoral factors, and a substantial reduction in the nutrient intake or availability (5). Pancreatic exocrine insufficiency (PEI) represents an additional and fundamental cause of malnutrition in such clinical settings (6, 7), occurring when the exocrine pancreas is unable to maintain its normal digestive function (secretion of proteases, lipase, and amylase). It results in maldigestion and malabsorption of nutrients, which may be manifest as abdominal bloating or discomfort and changes in bowel movements. This condition may contribute to weight loss and malnutrition, despite intake meeting estimate caloric needs (4). Mechanisms of PEI involve disease- or treatment-related (e.g. postoperative changes in patients undergoing partial or total pancreatectomy) loss of pancreatic parenchyma and/or obstruction of the main pancreatic duct, which impede either the production of pancreatic enzymes or their transfer into the duodenum (8). In addition, enzyme function is dependent upon neutral luminal pH; the concomitant impairment of bicarbonate secretion from the pancreas further contributes to malabsorption due to the effects of unopposed gastric acid secretion (9).

Exocrine insufficiency is difficult to detect since testing for PEI is cumbersome (10, 11). Thus, PEI is often clinically assessed and empirically treated. Oral pancreatic enzyme replacement therapy (PERT) represents the standard treatment for PEI (10). Clinical evidence suggests that PERT may prevent weight loss and induce weight gain (8, 12, 13) and is associated with an improved quality of life and, possibly, survival in patients with advanced PDAC (8, 14, 15). However, data in this field are limited and heterogeneous (10). Notably, PERT is largely underused: a recent retrospective analysis showed very low rates of PERT prescription (21%) in patients with metastatic pancreatic cancer, even though most patients had tumors of the pancreatic head and were likely to have an obstructed pancreatic duct (16). Additionally, even when PERT is prescribed in patients presenting with PEI, its dosage is sub‐optimal in more than half of the patients (17).

We thus investigated the use of PERT and its impact on survival, body weight, and patient-reported maldigestion-related symptoms in patients affected by advanced PDAC undergoing first-line chemotherapy with gemcitabine plus *nab*-paclitaxel, in the context of a retrospective observational study conducted at two large PDAC referral Institutions in northern Italy.

## Materials and Methods

### Study Design and Patients’ Population

This observational retrospective study included patients with advanced pathologically confirmed PDAC, treated with gemcitabine plus *nab*-paclitaxel as first-line chemotherapy at the Section of Oncology of the University Hospital and Trust of Verona and at the Medical Oncology of IRCCS San Raffaele Scientific Institute, Milan, from March 2015 to October 2018. Inclusion criteria included: 1) age > 18 years; 2) histological diagnosis of PDAC; 3) stage III not amenable to upfront surgical resection or stage IV (according to American Joint Committee on Cancer TNM classification 8^th^ edition) scheduled for initiating first-line chemotherapy with gemcitabine plus *nab*-paclitaxel; 4) availability of data on PERT administration and outcomes of interest (survival; body weight; maldigestion-related symptoms); patients were further selected based on initiation and completion of their first-line treatment at the University of Verona, while patients treated at the IRCCS San Raffaele Scientific Institute of Milan received gemcitabine plus *nab*-paclitaxel in the control arm of PACT-19 phase II trial (18).

The analysis was approved by the local Ethics Committee (Prot. 1341 CESC). A retrospective analysis was performed on anonymized data and informed consent was not applicable. The study protocol conforms to the ethical guidelines of the 1975 Declaration of Helsinki as reflected in a prior approval by the institution’s human research committee.

### Outcomes of Interest

The primary outcome of the present study was to explore the relevance of PERT use in terms of overall survival (OS) in patients affected by advanced PDAC undergoing first-line chemotherapy. The OS was defined as the time from first-line chemotherapy initiation to the date of death for any cause or last follow-up. Secondary outcome included the impact of PERT on changes in body weight and maldigestion-related symptoms after three months from the start of chemotherapy.

### Assessments

Data on patients’ diagnosis and treatment were retrieved from patients’ files. Demographics and clinical data evaluated included age, gender, Eastern Cooperative Oncology Group (ECOG) performance status, tumor site (pancreatic head, body, or tail), tumor size at diagnosis, and tumor stage (locally advanced unresectable or metastatic disease) at the time of first-line chemotherapy initiation and the presence of liver metastasis, baseline carbohydrate antigen (CA) 19-9 and fasting glucose levels.

Surgery of the primary and administration of scheduled chemotherapy (gemcitabine plus *nab*-paclitaxel) were recorded, too. During treatment, patients were also assessed for weight changes and the presence of patient-reported gastrointestinal symptoms by a questionnaire. Subjects were asked to indicate the presence or absence of symptoms that could be attributable to maldigestion (appetite loss; feeling of indigestion; bloating; frequent stools; floating or greasy/fatty in stool). Regarding PERT, the composition and the daily enzyme dose (recalculated as the number of capsules containing 10,000 U.Ph.Eur. of lipase) were evaluated. Patients were classified as PERT-treated if they were already initiated on PERT at the beginning of the first-line chemotherapy, or began PERT concurrently (i.e., within a week of the chemotherapy initiation).

### Statistical Analysis

All data were recorded in a dedicated electronic, anonymized CRF, with each patient assigned an alphanumeric code. Descriptive data are presented as median with range for continuous variables, frequencies and percentages are reported for categorical variables. Continuous baseline characteristics were compared between subgroups using the independent samples t-test or Mann-Whitney U-test depending on their distribution. Categorical variables were compared using the chi-square test. Follow-up was reported according to Shuster et al. The hazard ratios (HR) and the 95% confidence interval (CI) were estimated for each variable using the Cox model. The included variables in the univariate analysis for OS were age, gender, ECOG performance status, tumor location, tumor size at diagnosis, stage, presence of liver metastasis, surgery of the primary, Ca 19-9, fasting glucose levels, unintentional weight loss in the previous 6 months, the presence of maldigestion-related symptoms, body weight change (dichotomized as weight gain < 2% or ≥ 2% after three months from the start of first-line chemotherapy, according to a cut-off value established in a previous analysis from our institution (19) and PERT use (dichotomized as “yes” or “no”).

A multivariate Cox proportional hazard model with clinical, pathological and nutritional factors was developed using the stepwise regression (forward selection, enter limit and remove the limit, p=0.10 and 0.15, respectively) to identify independent predictors of OS. Harrell’s guidelines for the identification of the correct number of covariates were taken into account for the power analysis. Outcomes were estimated by the Kaplan–Meier product limit method. The log-rank test was used to assess differences between subgroups. Associations between variables were analyzed according to the Pearson chi-square test for categorical variables and the *t*-test for continuous variables. Significance was defined at p<0.05. The SPSS (V.18.0), R (V.2.6.1) and MedCalc (V.14.2.1) licensed statistical programs were adopted for all analyses.

## Results

### Baseline Patients’ Population Characteristics

**Figure 1:**
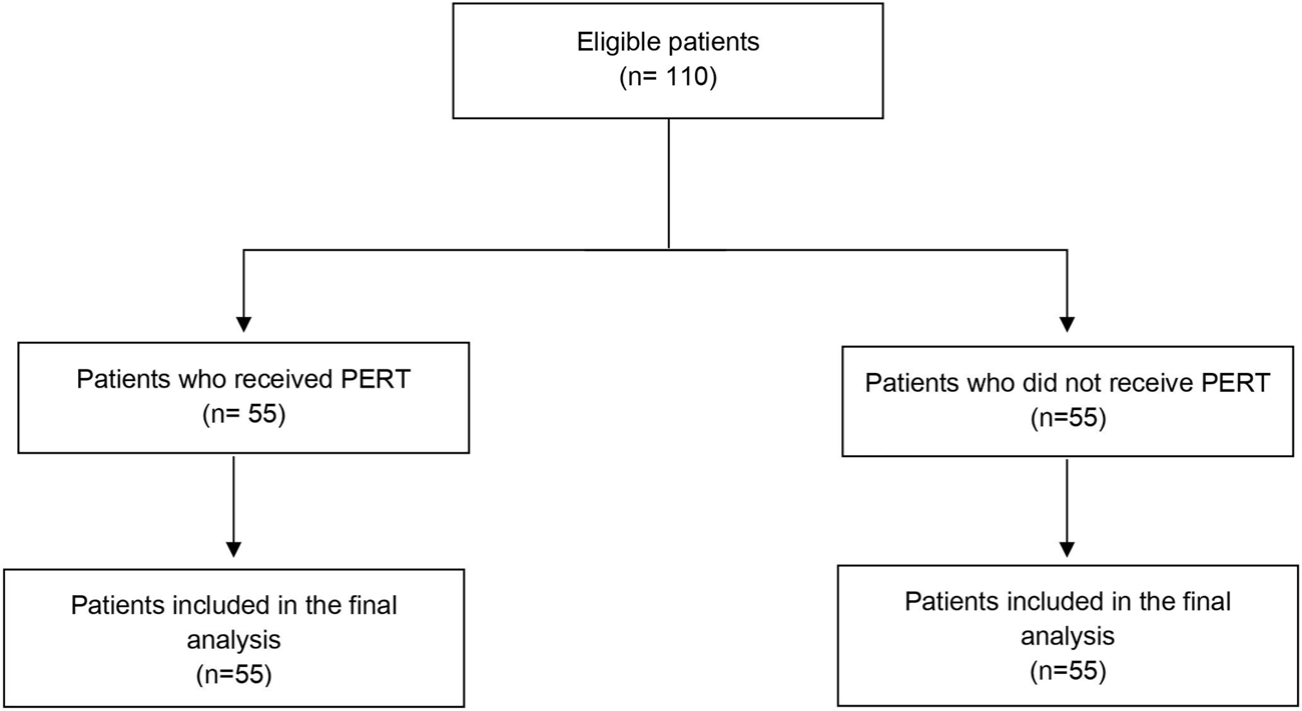
Flow chart of the studied subjects. PDAC, Pancreatic ductal adenocarcinoma; PERT, Pancreatic Enzyme Replacement Therapy.

The flow diagram of the patients included in the study is presented in **Figure 1**. Overall, 110 patients were included in the study. Patients’ characteristics are detailed in **Table 1**. Twenty-four patients (21.8%) had locally advanced and 86 (78.2%) metastatic disease; the tumor was more frequently located in the pancreatic head (81.8% of the cases) rather than in the body-tail (18.2% of the cases); 17 patients (15.5%) had received previous surgical resection of the primary, of which 15 (88.2%) had undergone resection of the pancreatic head. Median follow-up was 12 months (range 2-55 months).

**Table 1:** Baseline patients’ characteristics, according to Pancreatic Enzyme Replacement Therapy.

Variable	All (110 patients)	PERT (55 patients)	No PERT (55 patients)	*p-value*
**Male, N (%)**	53 (48.2)	27 (49.1)	26 (47.3)	*0.85*
**Median Age (years) [range]**	65 [37-81]	66 [37-78]	64 [37-81]	*0.93*
**Performance Status (ECOG), N (%)**				*0.09*
0-1	91 (82.7)	49 (89.1)	42 (76.4)	
2-3	19 (17.3)	6 (10.9)	13 (23.6)	
**Tumor location, N (%)**				*0.62*
Head	90 (81.8)	46 (83.6)	44 (80.0)	
Body-tail	20 (18.2)	9 (16.4)	11 (20.0)	
**Median tumor size at diagnosis [range] (cm)**	3.7 [1.2-8]	3.5 [1.3-8]	3.8 [1.2-7.5]	*0.43*
**Tumor Stage, N (%)**				*0.12*
Locally advanced unresectable	24 (21.8)	14 (25.5)	10 (18.2)	
Metastatic	86 (78.2)	41 (74.5)	45 (81.8)	
**Previous surgical resection of the primary, N (%)**				*0.19*
Yes	17 (15.5)	11 (20.0)	6 11.0)	
No	93 (84.5)	44 (80.0)	49 (89.0)	
**Median Ca 19.9 [range]**	382 [0.6-26800]	289 [0.9-23000]	390 [0.6-26800]	*0.27*
**Median fasting glucose levels (mg/dl) [range]**	109 [68-284]	112 [68-281]	114 [81-284]	*0.33*
**Unintentional weight loss ≥10%, N (%)**				*0.87*
Yes	65 (59.1)	32 (58.2)	33 (60.0)	
No	45 (40.9)	23 (41.8)	22 (40.0)	
**Maldigestion-related symptoms**				
Appetite loss	86 (78.2)	40 (72.7)	46 (83.6)	*0.17*
Feeling of indigestion	85 (77.3)	44 (80.0)	41 (74.5)	*0.50*
Bloating	105 (95.5)	54 (98.2)	51 (92.7)	*0.17*
Frequent stools	87 (79.1)	46 (83.6)	41 (74.5)	*0.24*
Floating or greasy/fatty in stool	84 (76.4)	44 (80.0)	40 (72.7)	*0.37*

ECOG, Eastern Cooperative Oncology Group; PERT, Pancreatic Enzyme Replacement Therapy.

Sixty-five (59.1%) showed significant weight loss (≥10% of their usual body weight within six months from diagnosis). According to cancer site, a significant weight loss was observed in 58 (64.4%) of 90 patients with pancreatic head involvement and in 12 (60%) out of 20 patients with body-tail cancer, respectively (*p=0.80*). Most patients reported the presence of maldigestion-related symptoms at diagnosis as shown in **Table 1**. Such symptoms were not restricted to patients with pancreatic head tumors: appetite loss, feeling of indigestion, and bloating were reported at similar frequencies by patients with head or body/tail primaries; however, frequent and floating or greasy/fatty stools were reported significantly more frequently by patients with a pancreatic head primary (83.3% versus 60%, *p=0.03*, and 81.1% versus 55%, *p=0.02*, respectively) (**Supplementary Table 1**). PERT was administered in 55 patients (50%). Enteric-coated, porcine enzyme preparations were used in all patients analyzed. The reported median enzyme dose was 80,000 U.Ph.Eur of lipase per day; 23 patients (41.8%) received 70,000 U.Ph.Eur of lipase per day or fewer. Among patients with baseline maldigestion-related symptoms, only 43 (50.6%) received PERT. According to tumor location, 46/90 (51.1%) patients with tumors in the pancreatic head and 9/20 (45%) patients with disease in the body-tail received PERT. Only 66.7% of patients who underwent previous pancreaticoduodenectomy were given PERT, although all of them reported signs or symptoms of maldigestion. Overall, there were no significant differences in baseline characteristics between patients who received or did not receive PERT (**Table 1**).

### Survival Outcomes and Multivariate Analysis

Median OS for the entire group was 12 months (95% CI 9-15). Overall, 1- and 2-year OS rates were 50% and 15.1%, respectively. At multivariate analysis, previous surgical resection of the primary, weight gain ≥2% after three months from the start of first-line chemotherapy and PERT use were independent predictors of OS (**Table 2**), regardless of the dosage of enzymes received.

**Table 2:** Independent predictors of overall survival at multivariate analysis.

Variable	HR	95% CI	*p-value*
**Previous surgical resection of the primary** (*No vs Yes*)	2.67	1.25 - 5.69	*0.011*
**Weight gain** *(<2% vs ≥2%)*	1.68	0.96 - 2.93	*0.07*
**PERT use** *(No vs Yes*)	2.85	1.67 - 4.86	*<0.001*

HR, Hazard Ratio; CI, confidence intervals; vs, versus; PERT, Pancreatic Enzyme Replacement Therapy.

**Figure 2:**
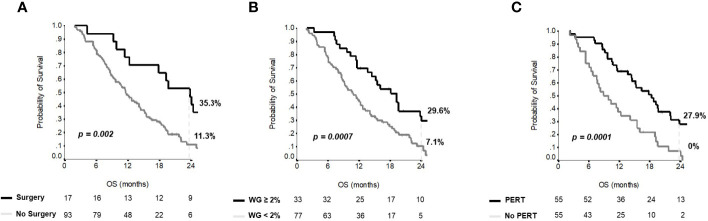
Overall survival curves for independent variables at multivariate analysis. Kaplan-Meier curves for **(A)** overall survival according to surgery; **(B)** overall survival according to weight gain; **(C)** overall survival according to PERT administration.

**Figure 2** shows the Kaplan–Meier curves for OS at 24 months according to surgery, weight gain and PERT administration. No interactions between PERT and either previous surgery (*p=0.475*) or weight gain at three months (*p=0.696*) were observed.

### Effect of PERT Administration on Weight Changes and Maldigestion-Related Symptoms

**Figure 3:**
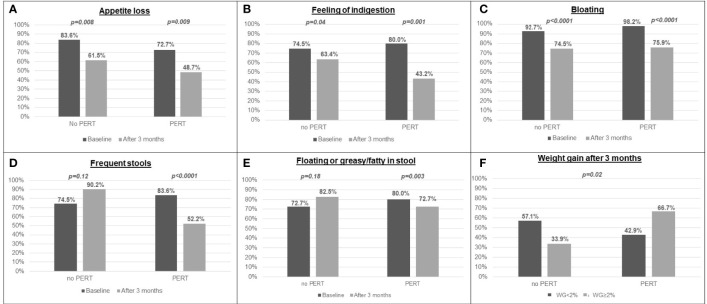
Prevalence of maldigestion-related symptoms and weight gain after 3 months in patients who were prescribed PERT and in patients who did not: **(A)** appetite loss; **(B)** feeling of indigestion; **(C)** bloating; **(D)** frequent stools; **(E)** floating or greasy/fatty stools; **(F)** weight gain.

As shown in **Figure 3**, maldigestion-related symptoms, such as appetite loss, feeling of indigestion and bloating were reported significantly less frequently after three months of gemcitabine/*nab*-paclitaxel treatment by both PERT-treated and -untreated patients, even though such improvement was more pronounced in patients who received PERT. Conversely, symptoms such as frequent and floating or greasy/fatty stools significantly decreased only in patients receiving PERT in addition to chemotherapy (*p<0.0001* and *p=0.003*, for frequent and floating or greasy/fatty stools, respectively; **Figure 3**). Notably, the improvement in symptoms after 3 months significantly correlated with daily enzyme dose (*p ≤ 0.0001*): 17 of 31 patients (54.8%) who took more than 80,000 U.Ph.Eur of lipase per day did not report gastrointestinal symptoms, whereas all the 23 patients (100%) who took < 80,000 U.Ph.Eur of lipase per day reported maldigestion -related symptoms.

Most importantly, after three months from the start of first-line chemotherapy, only 33.9% of patients receiving chemotherapy alone reported an increase in body weight ≥2%, as compared to 66.7% of patients receiving PERT in addition to chemotherapy (*p=0.02*; **Figure 3**); differently from maldigestion-related symptoms, increase in body weight was not significantly associated with the daily enzyme dose received (*p=0.65*).

## Discussion

In the present retrospective analysis, PERT was prescribed to only 50% of advanced PDAC patients, regardless of primary tumor location, previous surgery, or presence of maldigestion-related symptoms; moreover, enzyme dosage would be considered suboptimal, according to current recommendations (10), in the majority of patients who did receive PERT. Most importantly, our study shows that PERT administration is associated with a significant increase in body weight and longer OS, regardless of the dosage of enzymes received, while alleviation of maldigestion-related symptoms requires adequate enzyme intake levels.

Approximately, 80% of patients with PDAC present with either locally advanced or metastatic disease at diagnosis (1). In this clinical setting, the assessment of patients’ general conditions and supportive care needs holds a crucial role, in order to make chemotherapy possible and tolerable, thus prolonging survival (20). As the prevalence of nutritional derangements in patients with pancreatic cancer is among the highest in solid tumors, a multidisciplinary nutritional management is receiving increasing attention as it may be crucial to optimize treatment tolerance and efficacy, but it is not yet part of the routine care (21).

At presentation, the vast majority of patients of our cohort reported a significant unintentional weight loss and had symptoms that could be associated with maldigestion, in line with previous data (22). Maldigestion-related symptoms were not restricted to patients with the tumor located in the head of the pancreas. However, the prevalence of frequent and floating or greasy/fatty stools, specific PEI-related symptoms, was significantly higher in this subgroup likely due to the obstruction of the main pancreatic duct. Nevertheless, about half of them did not receive PERT. Lack of PERT was also frequent in patients who had previously undergone pancreaticoduodenectomy.

Consistent with these observations, a retrospective study by Landers et al., including 129 patients with metastatic PDAC, reported that only 21% received PERT prescription, despite the fact that more than 70% had gastrointestinal symptoms, like abdominal pain, meteorism, and steatorrhea (16). One of the reasons for this notable gap in care may be due to the lack of established clear guidelines for PERT administration and of evidence for clinical improvement in outcomes in PDAC patients. In this regard, a panel of experts in this field recently suggested that empiric treatment with PERT should be started, without prior testing, in all patients with a pancreatic head resection or tumor in the head of the pancreas and when there is a clinical suspicion of PEI, based on the typical symptoms and signs of malabsorption and malnutrition. Moreover, they suggested an initial dose of 40,000-50,000 U.Ph.Eur of lipase per meal and 25,000 U.Ph.Eur per snack (10). Considering these recommendations, our results showed that most patients do not take adequate enzyme supplementation, confirming previous data (17).

The most interesting result of our analysis is represented by the potential prognostic role of PERT in terms of survival in advanced PDAC patients undergoing first-line chemotherapy. Indeed, we found that PERT administration was a significant independent predictor for longer OS (regardless of the dose of enzymes received), in addition to other known independent prognostic factors, such as previous surgical resection of the primary and on-treatment weight gain (19). There are several possible explanations for these results. It is plausible that untreated maldigestion determines a worse nutritional status, beyond the weight loss, which is associated with a decreased likelihood to complete planned chemotherapy in PDAC patients (23). Additionally, the presence of unspecific maldigestion-related symptoms may be confounded with chemotherapy side effects, leading to its interruption or modifications of its schedule. Unfortunately, the retrospective design of the present study does not allow the investigation of data about treatment interruption and/or dose reduction. A multicenter, prospective study investigating the association between the nutritional status, PEI, and use of PERT and dose-intensity of administered chemotherapy is currently ongoing and may answer these questions (24).

Intriguingly, PEI is a critical host factor in determining the intestinal microbiota composition (25), which can potentially modulate tumor sensitivity to therapeutic agents (26).

Benefits of PERT in PDAC patients were reported by a series of studies. A recent retrospective analysis by Roberts et al., including PDAC patients with different stages and treatments, observed that PERT was associated with a statistically significant survival advantage when compared with matched non‐PERT‐treated controls, despite the reported low usage of PERT (15). Recently, a systematic review and meta-analysis conducted by de la Iglesia et al. in patients with advanced PDAC found that six retrospective studies investigated the impact of PERT on survival (27). PERT administration was related to significantly longer survival as compared to no treatment, with a median OS difference of about four months. One study did not observe a statistically significant difference between PERT and placebo in terms of survival, but this may be attributed to the low dosage of PERT that was administered (from 6 to 9 tablets per day) and to the inadequate statistical power (28).

Besides, we found that patients under PERT reported a significant improvement in all maldigestion-related symptoms, with specific improvements in stool consistency and frequency, and weight gain, according to previous data (27). Of interest, some symptoms, such as appetite loss, feeling of indigestion, and, to some extent, weight loss improved while on chemotherapy treatment even without PERT administration, thus reinforcing the “palliative” impact of chemotherapy. This is in line with data supporting a relevant clinical benefit for chemotherapy in advanced PDAC patients, in terms of alleviation of pain, improvement of performance status, and weight gain (29, 30); as confirmed by the retrospective study by Kim et al., these improvements in clinical outcomes correlate with OS and PFS, but not necessarily with a radiological response (31). However, some symptoms, namely frequent and floating or greasy/fatty stools, only improved and weight gain was significantly more frequent when PERT at optimal daily enzyme doses was added to standard chemotherapy.

Overall, most evidence regarding PERT use derives from small studies, characterized by considerable heterogeneity, in terms of disease stage and treatments received. In this view, strengths of the present study include the homogenous cohort of patients affected by advanced PDAC, all treated with the same first-line chemotherapeutic regimen. Also, patients were treated in two high-volume academic hospitals with appropriate expertise in multidisciplinary PDAC management, which it has been associated with improved clinical outcomes in this field (32).

The retrospective design, however, is the main limitation of the present study; the analysis is limited to hard objective variables that were well-recorded in clinical practice. A reverse causation bias for which patients with a likely better prognosis or engagement in the treatment plan were more likely to receive PERT cannot be fully excluded in this setting. Moreover, in the present study, the prevalence and severity of PEI were not investigated and thus it is unclear what proportion of patients under PERT or untreated had PEI. Anyway, the observed use of PERT (50%) suggests that a large proportion of patients were undertreated, even in high-volume Centers, given the expected prevalence of PEI in this clinical setting (72%) (27).

Besides, fecal elastase (FE-1), the most commonly used test for pancreatic exocrine function, has a documented limited accuracy and usefulness in advanced PDAC (33). Thus, the use of this analysis in a cohort of patients with a very high pre-test probability would not lead to an increased rate of diagnosis of PEI. Additionally, it could even lead to false-negative results: in patients affected by advanced PDAC in the head of the pancreas (most of our cohort) a value of FE-1< 200 µg/g would only increase the probability of PEI from 87% to 90% (10). In this scenario, it is considered correct to start the treatment always, or at least in case of impaired nutritional status or symptoms of PEI, shown by the vast majority of our patients (**Table 1**).

Another limit may be related to the patients’ selection. Indeed, at the University of Verona patients were selected on the basis of initiation and completion of their first-line treatment, while patients treated at the IRCCS San Raffaele Scientific Institute of Milan received gemcitabine plus nab-paclitaxel in the control arm of the PACT-19 phase II trial. This could have generated a selection bias, as patients included in the study could be more fit individuals compared to the general pancreatic cancer population.

**Figure 4:**
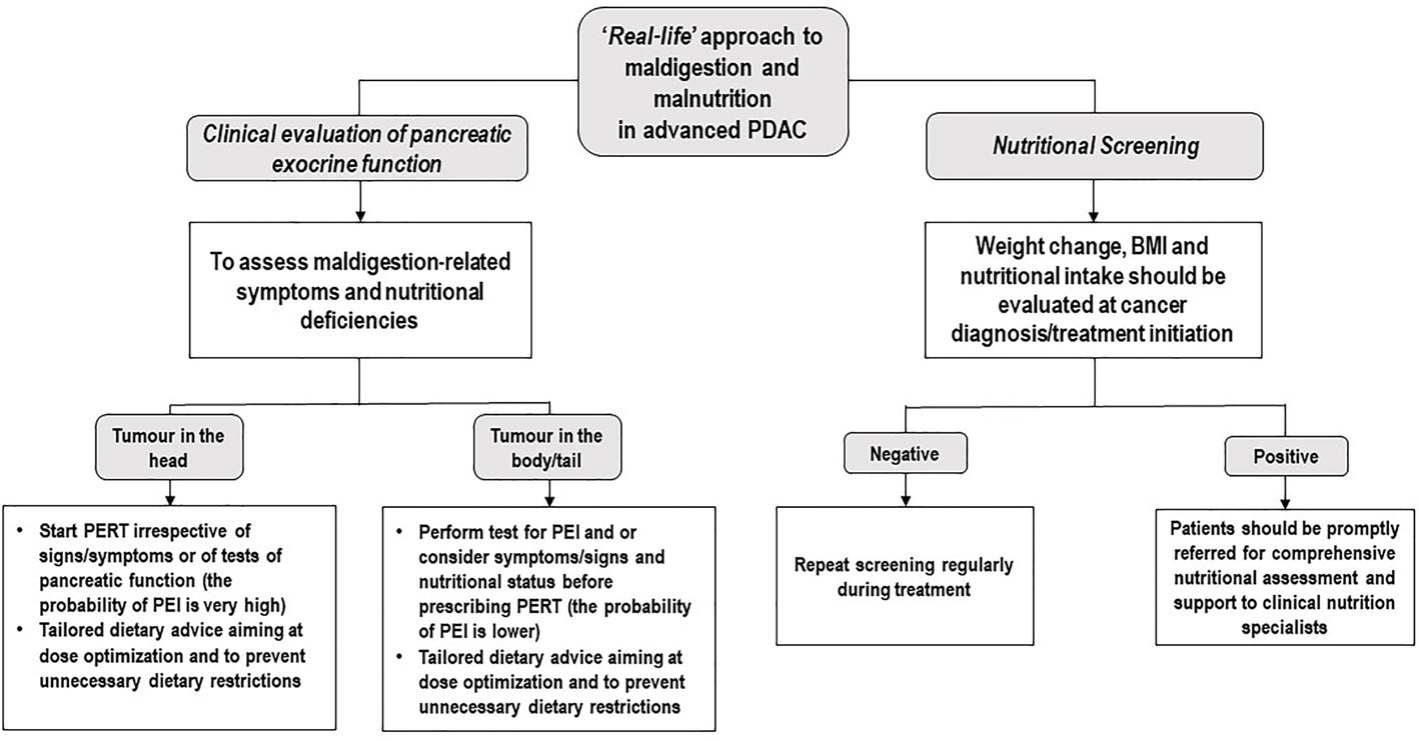
Potential ‘*Real-life*’ approach to maldigestion and malnutrition in advanced PDAC. PDAC, Pancreatic ductal adenocarcinoma; PERT, Pancreatic Enzyme Replacement Therapy; PEI, Pancreatic exocrine insufficiency; BMI, Body Mass Index.

In summary, the present study confirms a general lack of awareness about PERT among oncologists taking care of PDAC patients. Moreover, our data suggest that PERT administration may alleviate maldigestion-related symptoms, induce weight gain, and ultimately improve OS, in patients affected by advanced PDAC undergoing standard first-line chemotherapy with gemcitabine plus *nab*-paclitaxel. It is imperative to bring awareness to the gap between recent recommendations on PEI evaluation as well as its treatment in clinical practice. PERT use and dose optimization, together with personalized dietary counselling aiming at teaching patients the principles of flexible dosing and preventing them from adopting unnecessary dietary restrictions, should be integrated into early supportive care next to nutritional risk screening (**Figure 4**) as part of the best standard of PDAC treatment.

## Data Availability Statement

The raw data supporting the conclusions of this article will be made available by the authors, without undue reservation.

## Ethics Statement

The studies involving human participants were reviewed and approved by the local Ethics Commitee. The ethics committee waived the requirement of written informed consent for participation.

## Author Contributions

IT: Study design, data collection, data interpretation, writing–original draft, writing–review and editing. LC: Study design, data collection, data interpretation, writing–review and editing. UP: Data collection. IS: Statistical analysis. AC: Data collection, writing–review and editing. DT: Editing and critical appraisal of content. AAv: Data quality and statistical analysis. AAu: Editing and critical appraisal of content. GO: Data collection. SP: Editing and critical appraisal of content. LF: Editing and critical appraisal of content. GC: Contributions to the original draft, editing and critical appraisal of content. EB: Contributions to the original draft. MR: Contributions to the original draft, review, and editing; verification of methods; and critical appraisal of content. GT: Editing and critical appraisal of content. MM: Senior author; contributions to the original draft, review, and editing; verification of methods; and critical appraisal of content. All authors contributed to the article and approved the submitted version.

## Conflict of Interest

The authors declared the following potential conflicts of interest with respect to the research, authorship, and/or publication of this article: IT reported speakers’ fees from Mylan and Fresenius Kabi. LC received honoraria or speakers’ fee from Novartis, Istituto Gentili and Eli-Lilly. SP received honoraria or speakers’ fee from Astra-Zeneca, Eli-Lilly, BMS, Boehringer Ingelheim, MSD and Roche. MR received travel expenses and personal honoraria for advisory boards from Celgene, Merck, Astra-Zeneca, Baxalta (2016), Baxter, Sanofi (2017), Servier, Shire, Eli Lilly, Pfizer (2016), Novocure (2016) and Novartis (2016), personal honoraria for steering committee work for AstraZeneca, and non-remunerated steering committee activities for Boston Pharmaceuticals. EB received speakers’ and travels’ fee from MSD, Astra-Zeneca, Celgene, Pfizer, Helsinn, Eli-Lilly, BMS, Novartis and Roche. EB received consultant’s fee from Roche, Pfizer. EB received institutional research grants from Astra-Zeneca, Roche. MM received speakers’ honoraria from and participated on advisory boards for: EUSA Pharma, Pfizer, MSD, AstraZeneca, Merck‐Serono and Mylan.

The remaining authors declare that the research was conducted in the absence of any commercial or financial relationships that could be construed as a potential conflict of interest.

## Publisher’s Note

All claims expressed in this article are solely those of the authors and do not necessarily represent those of their affiliated organizations, or those of the publisher, the editors and the reviewers. Any product that may be evaluated in this article, or claim that may be made by its manufacturer, is not guaranteed or endorsed by the publisher.
