# Supplementary material for: Pancreatic Enzyme Replacement Therapy in Patients Undergoing First-Line Gemcitabine Plus nab-paclitaxel for Advanced Pancreatic Adenocarcinoma
Source: Front Oncol. 2021 Sep 9;11:688889. doi: 10.3389/fonc.2021.688889 (PMC8458827; doi:10.3389/fonc.2021.688889)
Supplement: Supplementary file 1 [file Table_1.docx]

Supplementary Material

**Supplementary Table 1.** Prevalence of maldigestion-related symptoms at baseline, according to tumor location.

| **Maldigestion-related symptoms** | **All**  **(110 patients)** | **Head**  **(90 patients)** | **Body/tail**  **(20 patients)** | ***p-value*** |
| --- | --- | --- | --- | --- |
| Appetite loss | 86 (78.2) | 71 (78.9) | 15 (75) | *0.77* |
| Feeling of indigestion | 85 (77.3) | 71 (78.9) | 14 (70) | *0.39* |
| Bloating | 105 (95.5) | 85 (94.4) | 20 (100) | *0.58* |
| Frequent stools | 87 (79.1) | 75 (83.3) | 12 (60) | *0.03* |
| Floating or greasy/fatty stools | 84 (76.4) | 73 (81.1) | 11 (55) | *0.02* |

**
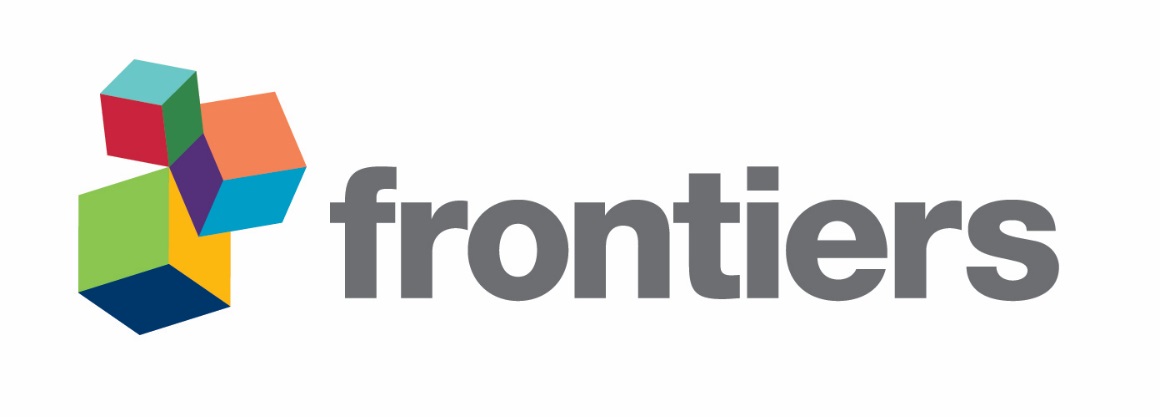
**
